# Dietary Habits of Insectivorous Bats (Family Hipposideridae) in The Rice Field

**DOI:** 10.21315/tlsr2025.36.3.13

**Published:** 2025-10-31

**Authors:** Nur Sakinah Humairah Zanarudin, Nurul Ain Elias, Suhaila Ab Hamid

**Affiliations:** School of Biological Sciences, Universiti Sains Malaysia. 11800 USM Pulau Pinang, Malaysia

**Keywords:** Bats, Diet, Diversity, Rice Fields, Pest Control, Kelawar, Diet, Kepelbagaian, Sawah Padi, Kawalan Perosak

## Abstract

This study establishes a foundational understanding of the dietary preferences of two insectivorous bats (Family Hipposideridae), *Hipposideros larvatus* and *Hipposideros cineraceus* caught in the rice field areas. The investigation focused on the analysis of their fecal pellets collected in areas near Gunung Keriang, Kota Setar, Kedah. A total of 40 pellets from eight individuals were meticulously examined. These eight bats were categorised into two distinct groups based on sex and reproductive stages (lactating and non-reproductive) from the two bat species. The dietary composition of *H. larvatus* comprised 55.2% Coleoptera, 23.2% Lepidoptera, 10.1% Hemiptera, 9.2% Diptera and 2.1% Hymenoptera. The diet of the bat species was significantly dominated by Coleoptera, accounting for over half of the overall dietary percentage. On the contrary, *H. cineraceus*, exhibited a different diet composition, with 68.0% Lepidoptera, 18.5% Coleoptera, 7.0% Diptera, 5.1% Hemiptera and 0.6% Hymenoptera. These variations in dietary preferences can be attributed to factors such as their differing abilities to digest chitin found on the elytra (forewing) of beetles, variations in size between the two species, distinct echolocation frequencies and differing reproductive states. Both *H. larvatus* and *H. cineraceus* have the potential to serve as effective pest controllers in rice fields by reducing insect pest populations, especially from the order Lepidoptera (rice stem borer) and Hemiptera (leafhoppers). Further research should be conducted in different locations to gain a more comprehensive understanding of these bat species’ diets and whether they exhibit exclusive or generalised feeding patterns.

## Abstract (fr)

**Abstrak:**

Kajian ini dijalankan untuk memahami pemilihan keutamaan pemakanan dua spesies kelawar pemakan serangga (Family Hipposideridae), Hipposideros larvatus dan Hipposideros cineraceus yang ditangkap di kawasan sawah padi. Kajian tertumpu pada analisis pelet najis kelawar yang dikutip di kawasan berhampiran Gunung Keriang, Kota Setar, Kedah. Sebanyak 40 biji pelet najis daripada lapan individu telah diperiksa dengan teliti. Lapan ekor kelawar ini dikategorikan kepada dua kumpulan berdasarkan jantina dan peringkat pembiakan (penyusuan dan bukan pembiakan). Komposisi pemakanan H. larvatus terdiri daripada 55.2% Coleoptera, 23.2% Lepidoptera, 10.1% Hemiptera, 9.2% Diptera dan 2.1% Hymenoptera. Pemakanan spesies kelawar didominasi dengan ketara oleh serangga daripada order Coleoptera, menyumbang lebih separuh daripada peratusan pemakanan keseluruhan. Sebaliknya, H. cineraceus, merekodkan komposisi diet yang berbeza, dengan 68.0% Lepidoptera, 18.5% Coleoptera, 7.0% Diptera, 5.1% Hemiptera dan 0.6% Hymenoptera. Variasi dalam keutamaan pemakanan ini boleh dikaitkan dengan faktor seperti kebolehan kelawar yang berbeza untuk mencerna kitin yang terdapat pada elytra (sayap depan) kumbang, variasi dalam saiz antara kedua-dua spesies, frekuensi ekolokasi yang berbeza, dan berbeza dalam keadaan pembiakan. Kedua-dua H. larvatus dan H. cineraceus berpotensi untuk berfungsi sebagai pengawal perosak yang berkesan di sawah dengan mengurangkan populasi serangga perosak terutamanya dari order Lepidoptera (pengorek batang padi) dan Hemiptera (kepinding). Kajian lanjut perlu dijalankan di lokasi lain untuk mendapatkan pemahaman yang lebih komprehensif tentang diet spesies kelawar ini, sama ada mereka mempamerkan corak pemakanan eksklusif atau umum.

**HIGHLIGHTS**

- Insects from the order Coleoptera (beetles), Lepidoptera (moths), Hemiptera (bugs), Diptera (flies) and Hymenoptera (ants, wasps and bees) are the dietary preferences of two insectivorous bats (Family Hipposideridae), *Hipposideros larvatus* and *Hipposideros cineraceus* caught in the paddy rice field areas.
- Coleoptera dominated the diet of the *Hipposideros larvatus*.
- *Hipposideros cineraceus* preferred a different diet composition, which was Lepidoptera.
- Bats have the potential to be a useful pest management tool in rice fields by controlling insect populations, particularly those insect pests from the Hemiptera and Lepidoptera orders.

## INTRODUCTION

Bats, members of the Chiroptera order, underwent significant reclassification during the 2000s, dividing into two main suborders: Megachiroptera and Microchiroptera. Megachiroptera lack echolocation abilities and predominantly do not consume insects, whereas Microchiroptera are insect-eating bats with the capacity for echolocation. Later, Microchiroptera was further categorised into two groups, Yangochiroptera and Yinpterochiroptera. Yangochiroptera essentially represents Microchiroptera without Rhinolophoidae, while Yinpterochiroptera encompasses two families, Pteropodidae and Rhinolophidae (Anderson & Ruxton 2020).

Different bat species occupy distinct ecological niches, leading to a wide range of dietary preferences, including insectivorous, frugivorous, nectarivorous, omnivorous, carnivorous, sanguivorous and piscivorous diets (Ingala *et al*. 2021). The specific types of insects that these bats consume depend on their habitats and environments. Studies on the diets of insectivorous bats reveal that the most consumed insects belong to the orders Lepidoptera (butterflies and moths), Diptera (true flies), Coleoptera (beetles) and Hemiptera (bugs). These bats can be categorised into four dietary groups: generalists, hard exoskeleton insect feeders, soft exoskeleton insect feeders and Lepidoptera specialists (Aguirre *et al*. 2003). A study done by Wijayanti *et al*. (2012) reported several insectivorous cave-dwelling bat species in the cave of Java, Indonesia, such as *Hipposideros sorenseni*, *H. bicolor* and *H. diadema*. Taray *et al*. (2021) also recorded bat species such as *H. coronatus* from a cave in the Philippines.

Several studies have investigated the diets of insectivorous bats, which analysed the dietary habits of bat species. Findings showed that common insects like Hemiptera and Coleoptera were part of the diet of tropical bats such as *Hipposideros armiger, Hypsugo cadornae, Rhinolophus thailandensis, Taphozous longimanus* and *T. melanopogon* (Weterings *et al*. 2015). Another study focused on the bat’s daytime diet, revealing that the most abundant orders in its diet were Hymenoptera, Coleoptera and Hemiptera (Vivas-Toro & Mendivil-Nieto 2022).

Bats are crucial in maintaining ecosystem health by controlling insect populations (Soliman & Emam 2022). However, many people remain unaware of their significance, leading to bat population declines. Specific bat species have the potential to act as insect pest controllers as an alternative approach to replace pesticide usage in rice fields. Identifying the insect groups eaten by bats in rice fields is essential for effective pest management. While various studies have explored bat diets using fecal analysis, there is a notable gap in research regarding bat preferences for insect groups. This study seeks to address this gap by focusing on insectivorous bats in rice fields, with objectives aimed at determining the insect groups present in bat feces and comparing the diets of two bat species within the rice fields.

## MATERIAL AND METHODS

The fieldwork for this research was conducted at a rice field near Gunung Keriang. Gunung Keriang, a limestone mountain formed over millions of years, is surrounded by rice fields and numerous caves, providing ideal habitats for bats. These caves serve as suitable roosting sites for bats, while the rice fields are abundant with various insect species, including bugs, beetles, moths, leafhoppers and other pests. These insects serve as a primary food source for the insectivorous bats of Gunung Keriang, making the area an optimal location for nocturnal foraging. To have the insect data that occupied the area, an insect trapping was conducted. The light trap was used to estimate the abundance and diversity of insect orders in the rice field. The light trap was put in three different sites around the rice field. The trapped insects were collected every hour from 2000 until 2200 and were placed into labelled plastic bags and preserved in the freezers for later identification. The traps may provide more realistic data on the insects available in that area, as the bats will forage over the area. After that, the insects were identified to the order level following Triplehorn and Johnson (2005).

It was hypothesised that bats roosted at Gua Gunung Keriang and foraged for insects in the vicinity of the Gunung Keriang rice fields. Harp traps were strategically positioned at four random locations within the rice field during the sampling occasions. The choice of varied and distant locations aimed to prevent bats from predicting trap placements and to increase their capture efficiency. Once captured, bats were carefully placed in cloth bags for further examination.

Physical measurements of each bat’s forearm, ear, body, tail, tibia, hindfoot and weight were taken to aid in species identification, as it relied on these measurements. The physical measurements of the bats were conducted and identified to species level based on Kingston et al. (2006) and Payne *et al*. (1998). A total of eight individuals (4 males and 4 females) comprising two species, *H. larvatus* and *H. cineraceus*, were captured. Bats were then held in individual cloth bags until they defecated (less than one hour), after which they were released back into the wild. The fecal pellets collected were kept in an Eppendorf tube. Insectivorous bats typically do not thoroughly chew their food and have rapid digestion. Five pellets from each individual were needed to calculate the average number of insects consumed. A total of 80 pellets for males and females of both bat species were used for this experiment. A piece of graph paper was cut to fit the bottom of the petri dish and secured with tape on the exterior. Numbers 1 through 5 were written at each of the graph paper’s four corners, and number 3 was written in the middle of the petri dish. The fecal pellet was then positioned at each number. Insect fragments found in the pellet were further examined under a microscope, with identification performed at the highest possible taxonomic level, typically the order level, following the taxonomic key from Triplehorn and Johnson (2005). The percentage frequency and volume of insect orders found were calculated using the formulas below from Zhang *et al*. (2005).


$$\begin{array}{l} \text{Percentage frequency of insect order}=\frac{\text{Total number of fragments of 1 order}}{\text{Total number of fragments}}\times 100\\ \text{Percentage volume of insect order}=\frac{\text{Grid occupied by 1 order}}{\text{Total number of grids}}\times 100 \end{array}$$

## RESULTS

The beetle or order Coleoptera was the most abundant insect order compared to other orders with a percentage of 71.88%, followed by Hemiptera with 23.83% and Hymenoptera (2.5%) (Table 1). The least order that was caught is dragonfly (order Odonata) with only 0.03%. Given that it is the most diversified group of insects, Coleoptera is expected to be the most abundant in comparison to other groups. Overall, the analysis of bats’ fecal pellets revealed the presence of five distinct insect orders: Coleoptera, Lepidoptera, Hymenoptera, Hemiptera and Diptera, in the diets of two bat species, *H. larvatus* and *H. cineraceus* (Table 2). Notably, *H. larvatus* exhibited a preference for Coleoptera, constituting 56.02% of its diet, followed by Lepidoptera (24.05%) and Hemiptera made up 9.51% of its consumed insects. On the other hand, *H. cineraceus* predominantly consumed Lepidoptera (69.5%), followed by Coleoptera (1.9%) and Hemiptera, making up 5.0% of its diet. Insects from the order Hymenoptera were reported to be the least consumed in both bat species, possibly due to insect habitat. However, there were no significant differences (*p* = 0.48) in the volumes of the insect orders for both bat species.

Male and female *H. larvatus* exhibited distinct dietary preferences, with females consuming Coleoptera at a higher volume of 53% compared to males at 43% (Table 3). Meanwhile, male *H. cineraceus* primarily favoured Lepidoptera, making up a substantial 83% of their diet, and their female *H. cineraceus* exhibited 75% for Lepidoptera. Notably, no significant difference between the sexes in both species, in *H. larvatus* and *H. cineraceus* (*p* < 0.05).

In the dietary preferences of lactating *H. larvatus*, Coleoptera accounted for the highest consumption at 61%, followed by Lepidoptera at 27%, Hemiptera at 6% and Diptera at 4% (Table 4). From observation during the sorting process, the fecal pellets produced by lactating females were found to be larger and contained more substantial fragments compared to those of non-reproductive females.

## DISCUSSIONS

Due to their diversity, some of them can be classified as scavengers, carnivores, parasites, or able to feed on both living and dead plants. As an alternative to controlling the population of pests, coleopterans can also serve as predators (Khan *et al*. 2021). Ladybird beetles belonging to the Coccinellidae family, for instance, consume eggs laid by rice stem borer (Ooi 2015). Hemiptera was discovered to be the second-largest insect group. According to Afifah and Sugiono (2020), Hemiptera, which primarily consisted of pests like brown planthoppers, green leafhoppers and leaf planthoppers, were the most common bugs found in rice fields. They are mostly sucking the rice plant’s sap or the paddy stem. On plant leaves, they were also observed to deposit their eggs (Ooi 2015). A similar finding was also reported in Thailand, where Hemiptera was found to be one of the most frequently caught insect species in rice fields (Thongphak & Iwai 2016).

The composition of insects found in the fecal samples of both bat species closely resembled findings from a study conducted by Weterings et al. (2015), in which *H. cineraceus* favoured Lepidoptera while *H. larvatus* showed a higher frequency and volume of Coleoptera consumption. This divergence may be attributed to the high prevalence of Lepidoptera in the local rice fields. Besides, major insect rice pests derive from the orders Lepidoptera and Hemiptera (Khan *et al*. 2021), which were the stem borers and armyworms (Lepidoptera) and leaf hoppers (Hemiptera). They occurred in high abundance during the vegetative and reproductive stages of the paddy (Salmah *et al*. 2020). Besides, insects from Lepidoptera, which are soft-bodied, were easier for *H. cineraceus* to consume and digest. The differences in dietary preferences may also be influenced by the echolocation calls of *H. cineraceus*. This aligns with research findings suggesting that bats consuming Lepidoptera, which was in this study, the moth, tend to be smaller and emit high-frequency calls, facilitating the detection of small, flying insects with loud noise (Waghiiwimbom *et al*. 2019). Insects from the order Coleoptera were reported to be consumed the most by *H. larvatus*. The abundance of Coleoptera in rice fields can be attributed to the ample food supply provided by these environments. Food availability (rice plants) contributes to a larger insect population, impacting their reproduction, growth, development, lifespan and overall fitness (Gutiérrez *et al*. 2020). Additionally, the two bat species might influence their dietary choices, as coleopterans could be too large for *H. cineraceus* to consume. Chitin content in Coleoptera forewing (elytra), contributing to their toughness, might also reduce bats’ digestibility (Murata *et al*. 2022).

Interestingly, female *H. larvatus* consumed more Coleoptera than males, marking a 10% difference in volume but fewer Lepidoptera. This variation might suggest that females are more active in foraging and consume a greater variety of insects compared to males. However, it’s important to note that the female tested in this study was non-reproductive, so the differences may not be solely attributed to gender but could also involve energy requirements for reproduction. Additionally, differences in body size between male and female *H. larvatus* could contribute to varying food intake, as larger individuals generally require more food to support their greater mass and metabolic demands (O’mara *et al*. 2016). Research on the common noctule bat, *Nyctalus noctula*, has shown that larger females have reproductive advantages, including better thermoregulation, higher milk quality and optimal timing for reproduction (Christe *et al*. 2007). Furthermore, the echolocation calls emitted by *H. larvatus* at 85 kHz (Thabah *et al*. 2006) may play a role, as larger insects produce more detectable noise, making them easier targets for bats.

It is anticipated that lactating females from both species would consume more food compared to their non-reproductive counterparts. Lactating females require additional energy (Altringham 2011) to sustain themselves and their offspring, as bats have six to nine weeks of pregnancy (Marnell *et al*. 2022). They care for their young by carrying them while flying, necessitating increased energy for feeding, milk production, and nursing. Lactating *H. cineraceus* females consumed more Lepidoptera than their non-reproductive females. Lactating female *H. cineraceus* consumed more moths, probably due to its limited foraging time. Barclay and Jacobs (2011) found that lactating females of Egyptian bats were away from their roost for only shorter periods. Therefore, lactating female *H. cineraceus* relies on the high number of food sources available in its surroundings, and in this study, it was the moth (Lepidoptera). A study by Kolkert *et al*. (2020) showed that higher attempts for Lepidoptera were recorded in bats foraging in crop areas. According to Pretzlaff *et al*. (2010), during periods of high energy demand, such as lactation and pregnancy, female bats must effectively regulate their body temperature to minimise energy expenditure. A study by Li *et al*. (2021) on *Vespertilio sinensis* (Asian particoloured bat) indicated that lactating individuals consume more Diptera and Coleoptera due to their higher protein content to meet their energy demands.

## CONCLUSION

This study offers insights into the dietary habits of two distinct insectivorous bat species in the Gunung Keriang area. There were nine insect orders captured in this area, with two insect orders (Lepidoptera and Hemiptera) that consisted of pests in rice fields. The research reveals that *H. larvatus* primarily consumes Coleoptera, while *H. cineraceus* predominantly feeds on Lepidoptera. These dietary preferences remain consistent across sexes and reproductive states within each bat species. This suggests that both *H. larvatus* and *H. cineraceus* have the potential to serve as effective pest controllers in rice fields by reducing insect pest populations, especially the rice stem borers (Lepidoptera). Further research is crucial to explore prey selection in these species across different locations. This will help to determine whether the diet composition observed in Gunung Keriang is specific to the area or a general trait of these bats. A broader understanding of their diets in various habitats will enhance knowledge of both similarities and differences between populations. Furthermore, improvements in the accuracy of insect species identification from bat feces can be achieved through molecular techniques.

## Figures and Tables

**TABLE 1:** Insect abundance during the sampling at Gunung Keriang.

Order	Abundance
Coleoptera	31,571 (71.88%)
Lepidoptera	178 (0.41%)
Diptera	304 (0.70%)
Hemiptera	10,467 (23.83%)
Odonata	12 (0.03%)
Orthoptera	163 (0.37%)
Hymenoptera	1,100 (2.50%)
Blattodea	81 (0.19%)
Dermaptera	38 (0.09%)

Total	43,914 (100.00%)

**TABLE 2:** Insect abundance during the sampling at Gunung Keriang.

Species	Lep	Col	Dip	Hym	Hem	Unide
*H. larvatus*	24.05 ± 1.94	56.02 ± 5.29	8.61 ± 2.29	1.54 ± 0.72	9.51 ± 2.37	0.27 ± 0.27
*H. cineraceus*	69.45 ± 8.93	17.86 ± 7.11	7.49 ± 2.95	0.59 ± 0.35	5.02 ± 0.66	0.67 ± 0.39

*Notes:* Lep = Lepidoptera, Col = Coleoptera, Dip = Diptera, Hym = Hymenoptera, Hem = Hemiptera, Unide = Unidentified.

**TABLE 3:** Diets comparison of the males and females from two bat species, *H. larvatus* and *H. cineraceus* in frequency (F) and volume (V).

Insect Order	*H. larvatus*				*H. cineraceus*			

	Male (*n* = 20)		Female (*n* = 20)		Male (*n* = 20)		Female (*n* = 20)	

	F (%)	V (%)	F (%)	V (%)	F (%)	V (%)	F (%)	V (%)
Lepidoptera	26.0	27.0	19.0	20.0	95.0	83.0	93.0	75.0
Coleoptera	49.0	43.0	66.0	53.0	3.0	10.0	2.0	9.0
Diptera	14.0	13.0	9.0	12.0	1.0	2.0	3.0	8.0
Hymenoptera	0.2	1.0	0.6	3.0	0.0	0.0	0.0	0.0
Hemiptera	10.0	15.0	5.0	12.0	0.5	4.0	1.0	7.0
Unidentified	1.0	1.0	0.0	0.0	0.7	1.0	0.3	1.0

**TABLE 3:** Diets comparison of the males and females from two bat species, *H. larvatus* and *H. cineraceus* in frequency (F) and volume (V).

Insect Order	*H. larvatus*				*H. cineraceus*			

	L (*n* = 20)		NR (*n* = 20)		L (*n* = 20)		NR (*n* = 20)	

	F (%)	V (%)	F (%)	V (%)	F (%)	V (%)	F (%)	V (%)
Lepidoptera	28.0	27.0	20.0	22.0	43.0	43.0	41.0	46.0
Coleoptera	66.0	61.0	66.0	57.0	40.0	39.0	6.0	15.0
Diptera	3.0	4.0	2.0	6.0	15.0	12.0	1.5	3.0
Hymenoptera	1.0	2.0	0.0	0.0	0.3	1.0	0.3	1.0
Hemiptera	3.0	6.0	2.0	5.0	1.5	5.0	1.0	5.0
Unidentified	0.0	0.0	0.0	0.0	0.0	0.0	0.0	0.0

*Notes:* L = Lactating, NR = non-reproductive, F = percentage frequency, V = percentage volume.
